# Electrodeposition and Optimisation of Amorphous Ni_x_S_y_ Catalyst for Hydrogen Evolution Reaction in Alkaline Environment

**DOI:** 10.1002/chem.202403030

**Published:** 2024-11-07

**Authors:** Cheng Lyu, Adeline Loh, Mikey Jones, David Trudgeon, Jack Corbin, Jianyun Cao, Zhenyu Zhang, Peter Connor, Xiaohong Li

**Affiliations:** ^1^ Renewable Energy Group, Department of Engineering Faculty of Environment, Science and Economy University of Exeter Penryn Campus Penryn TR10 9FE UK; ^2^ Camborne School of Mines, Department of Earth and Environmental Science Faculty of Environment, Science and Economy University of Exeter Penryn TR10 9FE UK; ^3^ Yunnan Key Laboratory of Electromagnetic Materials and Devices National Center for International Research on Photoelectric and Energy Materials, School of Materials and Energy Yunnan University Kunming 650091 P. R. China

**Keywords:** Hydrogen evolution reaction (HER), Nickel sulfide, Amorphous material, Cathodic electrodeposition, Anion exchange membrane (AEM) water electrolyser

## Abstract

Anion exchange membrane (AEM) water electrolysers have shown their potential in green hydrogen production. One of the crucial tasks is to discover novel cost‐effective and sustainable electrocatalyst materials. In this study, a low‐cost Ni−S‐based catalyst for hydrogen evolution reaction was prepared via a simple electrodeposition process from a modified Watts bath recipe. Physical characterisation methods suggest this deposit film to be amorphous. Optimisation of the electrodeposition parameters of the Ni_x_S_y_ catalyst was carried out using a rotating disk electrode setup. The optimised catalyst exhibited excellent catalytical performance in 1 M KOH on a microelectrode, with overpotentials of 41 mV, 111 mV and 202 mV at 10, 100 and 1000 mA cm^−2^ with Tafel slope of 67.9 mV dec^−1^ recorded at 333 K. Long‐term testing of the catalyst demonstrated steady performance over a 24 h period on microelectrode at 100 mA cm^−2^ with only 71 mV and 37 mV overpotential increase at 293 K and 333 K respectively. Full cell testing with the optimised Ni_x_S_y_ as cathode and NiFe(OH)_2_ as anode showed 1.88 V after 1 h electrolysis at 500 mA cm^−2^ in 1 M KOH under 333 K with FAA‐3‐30 membrane.

## Graphical abstract

This study reports the optimisation of nickel sulfide‐based catalyst prepared via electrodeposition process. Thiourea concentration and current density for deposition are the most influential factors. The optimised Ni_x_S_y_ catalyst shows excellent performance in an anion exchange membrane water electrolyser, achieving 500 mA cm^−2^ at a cell voltage of 1.88 V under 333 K in 1 M KOH.

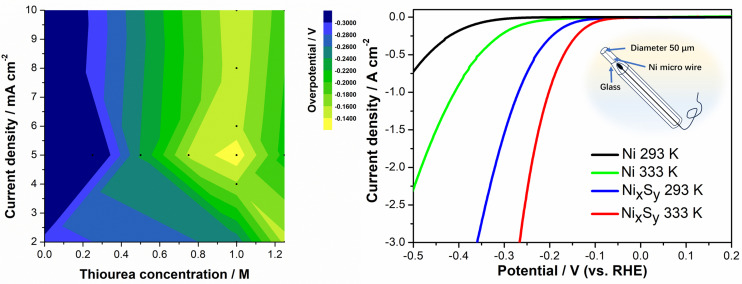

## 1 Introduction

Hydrogen is not only a vital feedstock material for industrial processes, but also a potential long‐term energy storage solution, allowing for the full exploitation of renewable energy sources as well as helping to balance the electricity grid. When coupled with renewable energy sources the splitting of water molecules via electrolysis produces carbon‐emission‐free green hydrogen, offering the potential to decarbonise key energy consumption sectors.[^1^, ^2^] However, roughly 99 % of hydrogen produced now is still fossil‐based, and low‐emission hydrogen accounts for less than 1 Mt (0.7 % of global production).^[3]^ Compared to traditional hydrogen production derived from fossil fuels, such as steam methane reforming which offers hydrogen production costs between $1.0 to $3.0/ ${kg}_{H_{2}}$ ,[^3^, ^4^, ^5^, ^6^, ^7^] the cost of green hydrogen produced via water electrolysis is currently too expensive.^[8]^ This is due to the energy and cost inefficiencies of the catalysts and electrode materials used in traditional alkaline water electrolysis (AWE) and proton exchange membrane (PEM) water electrolysis, which hinder the production of cheap green hydrogen.^[9]^ Water electrolysis involves two half reactions, the hydrogen evolution reaction (HER) and oxygen evolution reaction (OER), and catalysts play an important part in reducing the energy barriers of these two reactions thereby improving the efficiency of the system. To achieve cost competitive green hydrogen production, it is crucial to develop cheap and efficient HER and OER catalysts, ideally composed of earth abundant materials.[^10^, ^11^, ^12^, ^13^, ^14^] Anion exchange membrane (AEM) water electrolysers introduce the merit of the zero‐gap design, with minimum resistance through the system due to the replacement of the aqueous electrolyte with a solid polymer membrane, into an alkaline environment, which allows the operation at high current densities whilst application of cheaper, more abundant transition metal‐based catalysts.[^15^, ^16^] Its potential for seawater electrolysis also helps relieve the demand for fresh water as a feedstock for electrolysis.^[17]^

While platinum group metal (PGM) based catalysts are commonly recognised as the most active catalysts for HER in both acidic and basic environments,^[18]^ their high cost and relative scarcity make them financially unviable for large‐scale green hydrogen production. Nickel is a cheap and earth‐abundant alternative to PGMs, exhibiting good HER activity and corrosion resistance in an alkaline environment.^[15]^ Although the HER performance of nickel metal is inferior to that of PGM‐based catalysts,^[19]^ nickel‐alloys,^[20]^ sulfides,[^21^, ^22^, ^23^] selenides,^[24]^ hydroxides,[^25^, ^26^] phosphides,[^27^, ^28^, ^29^] and nitrides^[30]^ have all been shown to have significantly improved performance than nickel metal, which can be further boosted through modification with other transition metals.

Nickel sulfide has previously shown decent HER performance in alkaline environments during long‐term electrolysis.[^31^, ^32^] The first study on nickel sulfide as a HER catalyst dates back to the 1970s, where a Ni−S layer electrodeposited on a metal sheet demonstrated remarkable stability in an alkaline water electrolyser, surviving six months of continuous operation at a current density of 250 mA cm^−2^.^[31]^ Since then, studies on nickel sulfides such as Ni_3_S_2_, NiS and NiS_2_[^33^, ^34^, ^35^, ^36^] have emerged, some of which even report their use as a bifunctional catalyst for both HER and OER.[^37^, ^38^] There are several reported pathways for the synthesis of metal sulfide catalysts, such as chemical vapour deposition (CVD) methods,^[39]^ wet‐chemical methods,[^40^, ^41^] electrodeposition^[42]^ and hydrothermal methods.^[43]^ Each method offers various possibilities of altering the structural, chemical, and electronic properties of the metal sulfides materials, which grants great flexibility in controlling the properties for different purposes and applications. Among these preparation methods, electrodeposition has the advantage of not requiring high temperatures or large volumes of solvents that are necessary for hydrothermal, solvothermal and pyrolysis methods. However, systematic studies of the electrodeposition process of nickel sulfides as HER catalyst for alkaline environment, especially for AEM water electrolyser, is still limited. Parameters of the electrodeposition, such as the current density, the concentration of the active species in the electrolyte, as well as additives etc., have great impact on both the composition and morphology of the catalyst material, which affects the catalytic activity and stability of the material. The recipe for Ni_x_S_y_ electrodeposition in this study is based on the Watts bath, containing NiSO_4_, NiCl_2_ and H_3_BO_3_,which is used for nickel coating in industry.[^44^, ^45^, ^46^] NiSO_4_ is the primary source of Ni, while NiCl_2_ improves the conductivity and the deposition quality;^[45]^ H_3_BO_3_ is used as a pH buffer and thiourea as a source of sulfur. Although electrodeposition of nickel sulfide as a HER catalyst has been carried out in multiple studies, the mechanism for the cathodic deposition process has only been discussed in few publications.[^31^, ^42^, ^47^] Different from other sulfur sources, thiourea will simultaneously form complexes with Ni^2+^, such as [Ni(SC(NH_2_)_2_)_6_]^2+^,^[48]^ which is believed to be an intermediate involved in the deposition of nickel sulfide.

The use of electrodeposition for synthesis is also likely to result in amorphous materials, which are deemed to have a higher density of active sites due to their disordered structures.[^49^, ^50^, ^51^] For example, Benck et al.^[52]^ highlighted that the amorphous molybdenum sulfide they prepared had favourable surface properties, which contributed to its high activity and stability over extended reductive potential cycling. The amorphous features have adaptive catalytic performance to different conditions benefiting from its structural flexibility, whereas the active sites are usually located at the crystalline edges of materials with fixed crystal structure. Studies were also carried out on amorphous nickel sulfide materials as HER catalysts, which in some cases showed better catalytic performance than crystalline nickel sulfide materials.[^29^, ^53^]

Taking all of this into account, electrodeposited nickel sulfides were selected as a suitable HER catalyst candidate for further investigation due to their low cost, simple preparation, and potentially stable performance for AEM water electrolysis. In this study, the nickel sulfide based electrocatalysts were synthesised from a modified Watts bath using an electrodeposition method in order to encourage formation of amorphous structures. Systematic study of the effect of varying electrodeposition parameters was explored and further optimised using a rotating disk electrode (RDE) set‐up and the HER performance of the optimised Ni_x_S_y_ catalyst was then tested on a microelectrode setup. The electrodeposited catalyst properties were characterised by scanning electron microscopy (SEM), transmission electron microscopy (TEM), energy dispersive X‐ray spectroscopy (EDS), X‐ray diffraction (XRD) and X‐ray photoelectron spectroscopy (XPS) analysis, where co‐deposition of Ni and Ni−S was found. On top of this, as the long‐term stability of the catalyst is one of the most important design criteria for large‐scale electrolysis, long‐term stability testing of the optimised Ni_x_S_y_ catalyst was conducted in a microelectrode setup in 1 M KOH at temperatures of 293 K and 333 K, at atmospheric pressure for over 20 h. Full AEM cell testing with Ni_x_S_y_ cathode and NiFe(OH)_2_ anode showed 1.88 V after 1 h of electrolysis at 500 mA cm^−2^ in 1 M KOH with FAA‐3‐30 membrane. Compared to other works, this work not only reported an efficient PGM‐free HER catalyst but also collected data from full AEM cell electrolysis, which provides guidance for the practical application of this catalyst.

## Experimental Procedures

### Materials and Chemicals

Nickel (II) sulphate hexahydrate, NiSO_4_⋅6H_2_O (Fisher Scientific, ≥99 %), nickel (II) chloride hexahydrate, NiCl_2_⋅6H_2_O (Hogg laboratory supplies, ≥99.5 %), boric acid, H_3_BO_3_ (Sigma Aldrich, ≥99.5 %), thiourea, CS(NH_2_)_2_ (Hogg laboratory supplies, ≥99.5 %), potassium hydroxide, KOH (Sigma Aldrich, ≥90 %), hydrochloric acid, HCl (Sigma Aldrich, 36.5–38.0 %), sulfuric acid, H_2_SO_4_, (Sigma Aldrich, 90–100 %), Ni mesh (Dexmet 4Ni6‐050FA), 2‐propanol (IPA, ≥99.5 %, Sigma Aldrich). All chemicals were used as received. Deionised water (DI) from an Elga Biopure 600 was used in all experiments.

### Optimisation of the Electrodeposition of Nickel Sulfide

For the electrodeposition of catalyst samples on the RDE, a three‐electrode system was used in the RDE setup (AFMSRCE, Pine Research Instruments, USA), consisting of a glassy carbon RDE tip (ø=4 mm, Pine Research Instruments, USA) as the working electrode, Hg/HgO in 1 M KOH as reference electrode (fabricated in‐house) and Pt mesh (2×3 cm^2^) as the counter electrode. A 150 mL jacketed RDE cell (Pine Research Instruments, USA) was used for both deposition and testing. For microelectrode experiments, a Ni microelectrode (ø=50 μm) served as the working electrode, Ag/AgCl in 3 M NaCl and Hg/HgO in 1 M KOH were used as reference electrodes for electrodeposition and performance testing, respectively, with a Pt mesh as counter electrode. A small, jacketed glass cell of approximately 20 mL volume was used. Both the glassy carbon electrode and the microelectrode used in this study were well polished before and after each test using alumina slurries (Micro‐polish, Buehlar) of 0.3 μm and 0.05 μm particle sizes on microfibre cloth for 5 min with each slurry. DI water was used to clean the polished electrodes with assist of ultrasonic bath after each polishing.

The electrolyte for Ni_x_S_y_ deposition consisted of 100 mM nickel (II) sulphate hexahydrate and, 20 mM nickel (II) chloride hexahydrate as source of nickel, 20–80 mM of boric acid as the buffer to provide a steady pH environment and thiourea with concentrations between 0.25–1.25 M as a source of sulfur. To avoid the generation of possible impurities of NiO and Ni(OH)_2_ during electrodeposition, the pH of the electrolyte was altered to be between pH 3.5 and 4 by the dropwise addition of 1 M HCl into the solution. Cathodic deposition of the catalyst was conducted and recorded using chronopotentiometry (CP) with a potentiostat (BP‐300, BioLogic). Deposition was carried out using constant current densities between 1 mA cm^−2^ to 10 mA cm^−2^ over a range of 10–100 mins, before the electrodes were rinsed with DI and dried in air for testing in 1 M KOH.

### Electrochemical Measurements

Multiple electrochemical techniques were employed in this study using BioLogic potentiostat (BP‐300, Biologic) and EC‐lab software (version 11.33). Experiments in both rotating disk electrode (RDE) and microelectrode setups involved an initial electrodeposition step of the catalyst deposit from the prepared electrolyte followed by performance testing in 1 M KOH after the deposit was rinsed and dried. Samples deposited on the RDE during the recipe optimisation process were subjected to linear sweep voltammetry (LSV) from 0 V to 1.7 V (vs. Hg/HgO) at a scan rate of 20 mV s^−1^, followed by CP at −5 mA cm^−2^, −10 mA cm^−2^, −20 mA cm^−2^, −50 mA cm^−2^ and −100 mA cm^−2^ for 300 s, respectively. CP was used to ensure a more reliable estimation of the HER overpotential at an equilibrium. In the microelectrode setup, a customised glass cell with a water jacket was used, allowing the microelectrode to face upwards into the cell, preventing the accumulation of hydrogen bubbles generated during the test at the electrode surface. Control of the operating temperature to 333 K was achieved by a water bath (Grant TC120) pumping temperature‐controlled water through the water jacket of the cell, while there was no water passing through the water jacket for room temperature (293 K) tests. Due to the very small surface area of the microelectrode, a current as low as 19.6 μA can achieve a current density of 1 A cm^−2^, which minimises the influence of overpotential caused by ohmic drop existing in the system.

### AEM Cell Testing

The optimised catalyst was further tested in a 4 cm^2^ homemade AEM cell. The coating of the Ni mesh (Dexmet 4Ni6‐050FA) was carried out in a 150 mL beaker, containing the optimised solution with Ag/AgCl in saturated NaCl and Pt mesh as reference electrode and counter electrode respectively. The Ni mesh for coating was cleaned by ultrasonication in isopropanol and 1 M HCl for 5 min respectively ahead of the deposition process. A cathodic current density of 5 mA cm^−2^ over 20 mins was used to prepare the Ni_x_S_y_ coated Ni mesh. Solution containing 14.4 mM NiSO_4_, 3.6 mM FeSO_4_ and 25 mM (NH_4_)_2_SO_4_ was made for coating NiFe(OH)_2_, the deposition was carried out at a current density of 62.5 mA cm^−2^ over 200 s on Ni mesh.

Ni_x_S_y_ coated Ni mesh was assembled as cathode and NiFe(OH)_2_ coated Ni mesh anode in a zero‐gap AEM water electrolyser. Fumasep FAA‐3‐30 (thickness 30 μm) was chosen as the AEM for the test, pretreated by immersing the membrane in 1 M KOH for more than 72 hours with exchange of solution several times and heated to operating temperature (333 K) before assembly in the full cell setup.

During the operation of the cell, a water bath (Grant TC120) was set to maintain at 333 K with 1 L reservoir of 1 M KOH, and the heated solution was continuously pumped through the cell at a flow rate of 250 mL min^−1^ to serve as electrolyte by a peristaltic pump (Watson Marlow 323, UK). H_2_ or O_2_ bubbles generated at the electrodes are also washed away within the electrolyte.

### Structural and Morphological Characterisation

The morphology and composition of the deposited sample were analysed using FEG‐SEM‐EDS (FEI Quanta 650 FEG) and SEM‐EDS (TESCAN VEGA 3). To avoid the influence of the substrate, the Ni_x_S_y_ samples were prepared on either gold‐coated glass (Platypus technology) or polished carbon plates (FR10, SGL Carbon Bipolar Plates) for EDS analysis of Ni_x_S_y_. The surface area of the substrates was controlled by masking the electrode with non‐conductive tape. The samples were washed with DI water and dried in air before characterisation.

Transmission electron microscopy (TEM, JEOL 2100) was used to study the crystallisation and morphology of the electrodeposited sample. The sample was prepared by CP via deposition at −5 mA cm^−2^ for 1200 s on top of a polished carbon plate (FR10, SGL Carbon Bipolar Plates), and then exfoliated from the substrate with the assistance of a spatula. The crushed sample was then dispersed in isopropanol under ultrasonic vibration for 15 min. After the solution settled, a drop of liquid containing nanoparticles from the top transparent liquid layer was added to a holey‐carbon‐coated copper grid and air‐dried at room temperature. The dried copper grid with sample captured on top was then characterised by TEM.

X‐ray photoelectron spectroscopy (XPS, Thermo NEXSA) was used in this study to investigate the valance status and bonding information for the deposited thin film. Samples were prepared with carbon plates (FR10, SGL Carbon Bipolar Plates) as a conductive substrate, and the surface area was controlled to 1 cm^2^ by masking with non‐conductive tape before electrodeposition. The prepared samples were then delivered to Harwell XPS for testing using Thermo NEXSA XPS. The data analysis was then carried out on Casa XPS (version 2.1.0.1) software. The C 1*s* peak at 284.8 eV was selected to calibrate and compensate for the shift of the binding energy for all spectra.

## 2 Results and Discussion

### 2.1 Deposition Step Study

**Figure 1:**
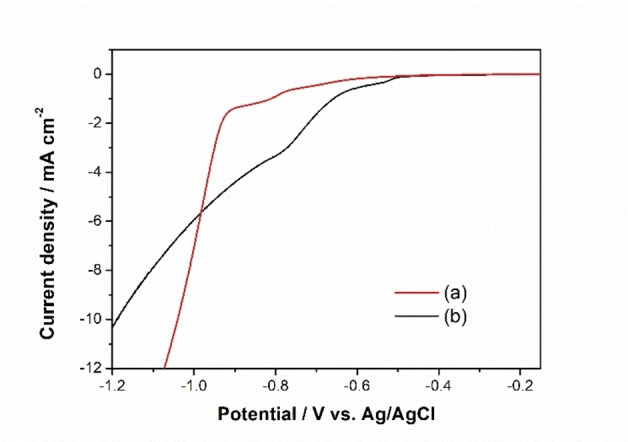
I−E curve recorded with linear sweep voltammetry method under scan rate of 10 mV s^−1^ with rotating speed of 2500 rpm in (a) 100 mM NiSO_4_, 20 mM NiCl_2_ and 60 mM H_3_BO_3_ solution, and (b) in 100 mM NiSO_4_, 20 mM NiCl_2_, 60 mM H_3_BO_3_ and 1 M CS(NH_2_)_2_; both solutions’ pH were controlled to 4 by adjusting with 0.01 M H_2_SO_4_.

To investigate the detailed electrodeposition process of Ni_x_S_y_, electrochemical tests on a RDE were performed. With help of RDE setup, investigation of the behaviour of steady state ions during the deposition process can be carried out, where the mass transport of ions is encouraged by mechanical convection. For the electrodeposition data, the potentials below are all vs. Ag/AgCl. Figure 1 shows the I−E curve obtained via LSV in range of −0.2 to −1.2 V with RDE spinning rate of 2500 rpm. In the solution only containing Ni^2+^ as active species, the deposition of Ni from Ni^2+^ (Equation (1)) can be found starting at around −0.60 V, and the main peak occurs at around −0.95 V. The standard potential for nickel deposition is −0.257 V vs SHE, and the Ag/AgCl vs SHE is +0.197 V under standard conditions (1 atm, 25 °C).^[54]^ According to the Nernst equation (Equation (S1)), the potential for Equation (1) is around −0.481 V at 25 °C in 100 mM NiSO_4_ solution. An overpotential for deposition of Ni is due to the application of glassy carbon as substrate, where extra nucleation energy is required compared to Ni metal substrates. After the addition of thiourea, the reduction peak at −0.56 V is enhanced due to the complex formation between Ni^2+^ and thiourea. Also the peak at −0.78 V is larger due to the deposition of nickel sulfide, from the complexes formed between Ni^2+^ and CS(NH_2_)_2_, which can be presented as [Ni(SC(NH_2_)_2_)_6_]^2+^ following the reaction shown in Equation (2). The deposit is a composite between nickel and nickel sulfide, here we use Ni_x_S_y_ as the name of the catalyst for convenience of expression.

The two‐step cathodic deposition of nickel sulfide is believed to follow the path shown in Equation (1) and (2). The acidic environment not only prevents the generation of nickel oxide and hydroxide during the deposition, but also prevents the hydrolysis of thiourea to give out sulfur ions, which could lead to the deposition of nickel sulfide without driving force of electricity by applying potential difference.

(1) $${Ni}^{2+}+{2e}^{-}=Ni$$

(2) $$x{\left[Ni{\left(SC{\left({NH}_{2}\right)}_{2}\right)}_{6}\right]}^{2+}+2xe^{-}+yH_{2}O={Ni}_{x}S_{y}+yOC{{(NH}_{2})}_{2}+\left(6x-y\right)CS({NH}_{2}{)}_{2}+{yH}_{2}\;$$

**Figure 2:**
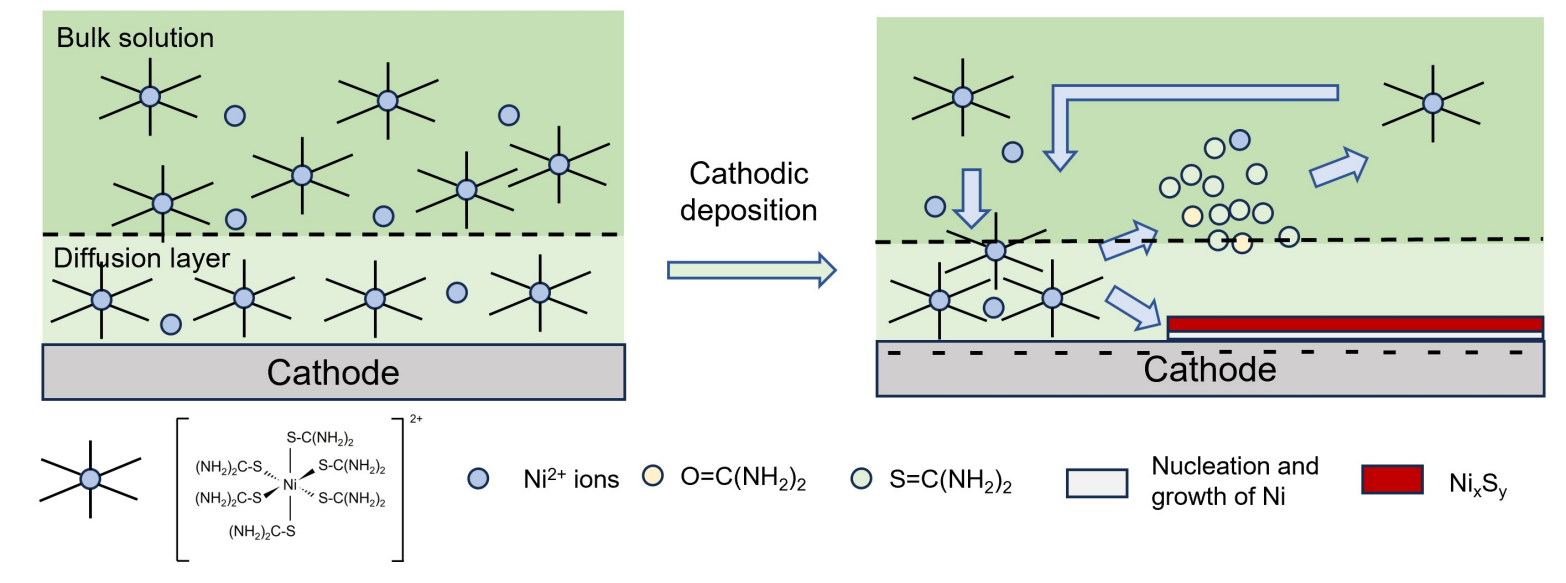
Illustration diagram for nickel sulfide deposition with thiourea as sulfur source.

From these steps, it is hypothesised that the cathodic electrodeposition of Ni_x_S_y_ (Equation (2)) occurs simultaneously with Ni deposition (Equation (1)), which results in a product containing a mixture of multivalence states between Ni^0^, Ni^+^ and Ni^2+^.[^31^, ^42^] The solubility product constant, K_sp_, of nickel sulfide, NiS, is very low, around 3×10^−19^ at 25 °C,^[55]^ meaning that it is insoluble and allows the simultaneous formation of nickel sulfide with the appearance of both Ni ions and S ions. However, no free S ion is released without electrochemical driving force. Figure 2 shows the deposition process of nickel sulfide with thiourea as sulfur source. Because the ratio between Ni and S is not 1 : 1, the electrodeposited samples are hence labelled as Ni_x_S_y_. Low current density conditions below 10 mA cm^−2^ were previously observed to induce the deposition of Ni_x_S_y,_ while higher current densities lead to Ni being the primary deposit,[^31^, ^56^] which is also suggested in Figure 1.

### 2.2 Optimisation of Ni_x_S_y_

#### 2.2.1 Rotating Speed

Since the RDE generates a steady state laminar flow at the surface through forced convection to improve the mass transport process as a hydrodynamic tool, it allows the study of steady state ions and can help control the IR drop during the tests by effectively removing the gas bubbles, it was chosen as the electrode for optimisation of the deposition of nickel sulfide.^[57]^ The kinetics of Ni^2+^ deposition was first studied. According to the Levich equation (Equation (3)), for the mass transfer‐controlled reactions, the current density should be in linear relation to the square root of the spinning rate.

(3) $$j_{L}=0.62\frac{nFD^{2/3}c}{\nu^{1/6}}\omega^{1/2}$$

**Figure 3:**
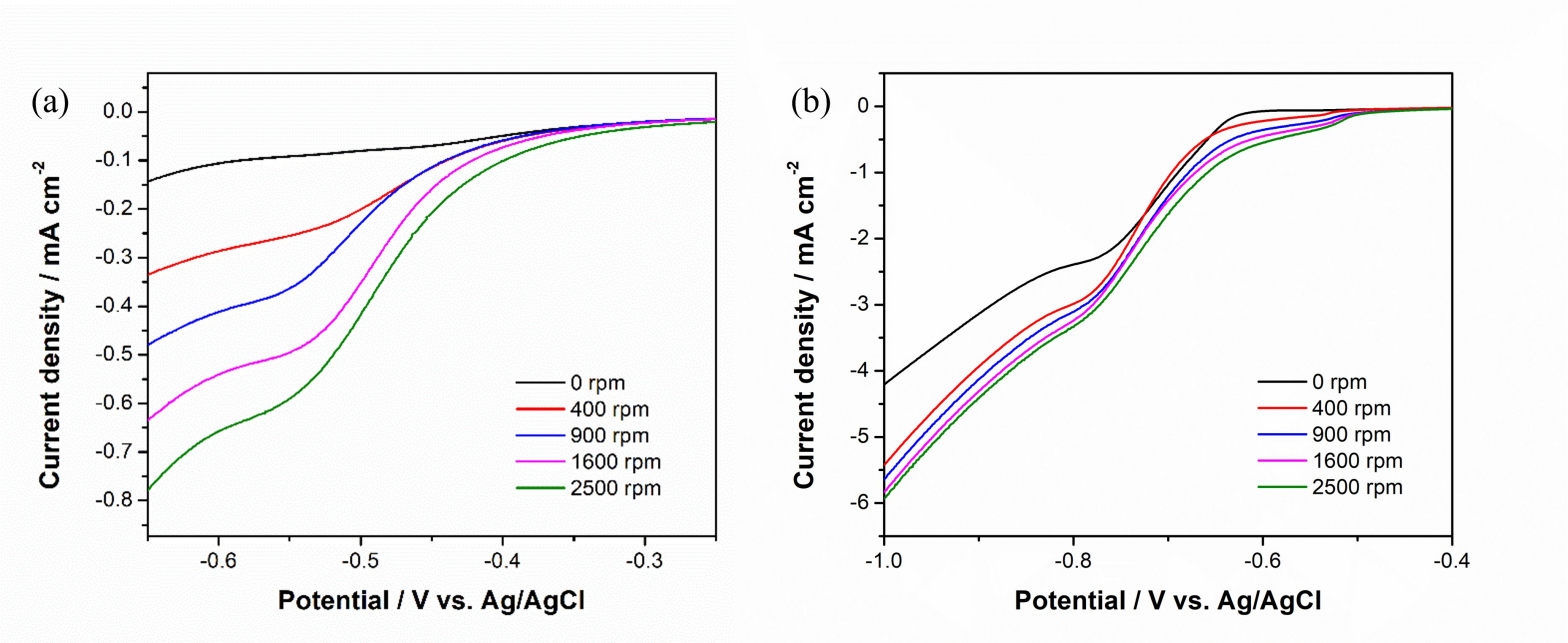
I−E curve recorded via LSV performed on GC RDE setup with scan rate of 10 mV s^−1^ at different rotating speed in (a) 100 mM NiSO_4_, 20 mM NiCl_2_, 60 mM H_3_BO_3_ and (b) 100 mM NiSO_4_, 20 mM NiCl_2_, 60 mM H_3_BO_3_ and 1 M CS(NH_2_)_2_; both solutions’ pH were controlled to 4 by adjusting with 0.01 M H_2_SO_4._

LSV was performed to study the deposition of Ni^2+^, as is shown in Figure 3(a). The rotational speed of the RDE, in rotations per minute (RPM), was varied to determine how it affects the mass transport of the reactants and products at the electrode surface during deposition and subsequent tests. The increase of current density can be observed with higher spinning rates, which indicates the process is mass transfer controlled. An overpotential due to the nucleation of Ni on GC can be observed as the deposition started at around −0.56 V but only after −0.9 V an increase of current density was found. After the addition of thiourea (Figure 3b), the reduction peak at −0.56 V is enhanced due to complex formation between Ni^2+^ and thiourea that leads to deposition of nickel sulfide, which showed linear change of current density to the square root of the spinning rate (Figure S1), while the peak at −0.78 V does not follow the same pattern. Comparing the deposition of nickel sulfide with and without the spinning of electrode, an increase of current density can be observed, but the current density does not keep growing linear to the square root of the spinning rate. Enhancement of mass transportation is beneficial for the deposition of both nickel and nickel sulfide.

**Figure 4:**
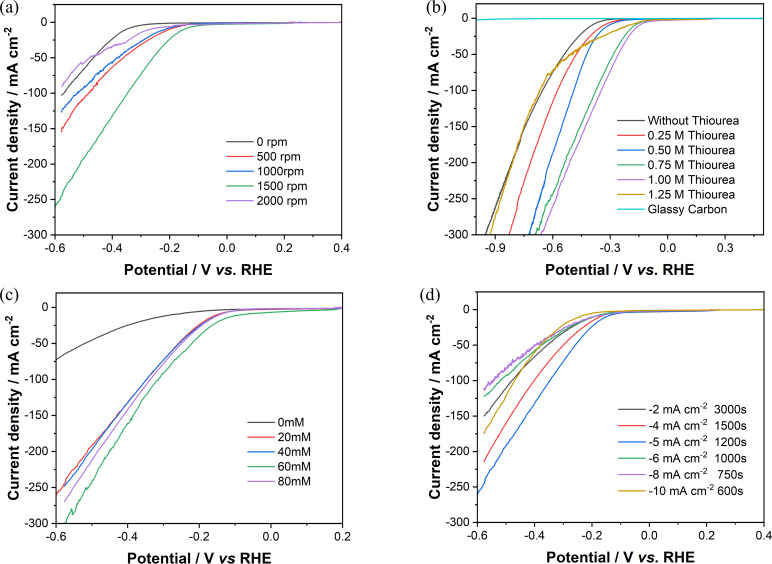
Plots of the I−V curves obtained via linear sweep voltammetry for catalysts deposited on glassy carbon RDE with different (a) different RDE rotational rates of RDE, (b) different thiourea concentrations, (c) different concentrations of boric acid as buffer and (d) different current density with same total electron transferred, during electrodeposition, tested in 1 M KOH solution under the room temperature, with the spinning rate of 1500 rpm during testing without IR compensation.

To investigate the influence of spinning rate of RDE during the deposition of nickel sulfide on the HER performance of the samples, catalysts were then prepared under a variety of spinning rates and tested in 1 M KOH. Figure 4(a) shows the HER performance of the catalysts recorded via LSV, where improvement of catalytic activity can be observed at higher rotational rates during deposition, benefiting from the enhanced mass transportation. However, the shearing force of the electrolyte on the deposit layer also increases with the increase in rotation speed and can be further amplified if the surface is rougher, compromising mechanical stability of the deposit. Detachment of the catalyst prepared at 2000 rpm was observed in the LSV as a sudden drop of current, and black particles were seen at the bottom of the cell. The catalyst deposited at 1500 rpm showed the lowest HER overpotential and therefore this rotational speed was selected as the optimal deposition condition for the following tests.

#### 2.2.2 Thiourea Concentration

Optimisation of the thiourea concentration in the deposition solution was then studied on RDE since this can alter the ratio of Ni to S in the electrodeposited nickel sulfide.[^31^, ^56^] Six different concentrations of thiourea were tried and corresponding LSV curves are shown in Figure 4(b). It can be seen that the deposited nickel sulfide samples showed a better HER performance than deposition without the addition of thiourea, which would lead the deposition to be just pure Ni metal. However, a high concentration of thiourea in the deposition electrolyte solution may also cause a less stable catalyst layer which is easily detached from the electrode during the HER test which was observable by eye. This was most obvious for the catalyst layer deposited using 1.25 M thiourea, the highest concentration tested. The concentration of the thiourea added for electrodeposition was demonstrated to be crucial for the formation of a highly active catalyst; when the thiourea concentration is 0.75 M or 1 M, the catalyst shows a noticeably lower overpotential than those obtained with lower thiourea concentrations on the RDE setup. Of the prepared catalysts, the sample synthesised with 1 M thiourea in the depositing electrolyte delivered the best HER catalytic performance, with overpotentials of 172 mV and 504 mV for HER of this sample at 10 mA cm^−2^ and 100 mA cm^−2^, respectively, in 1 M KOH without IR compensation.

**Figure 5:**
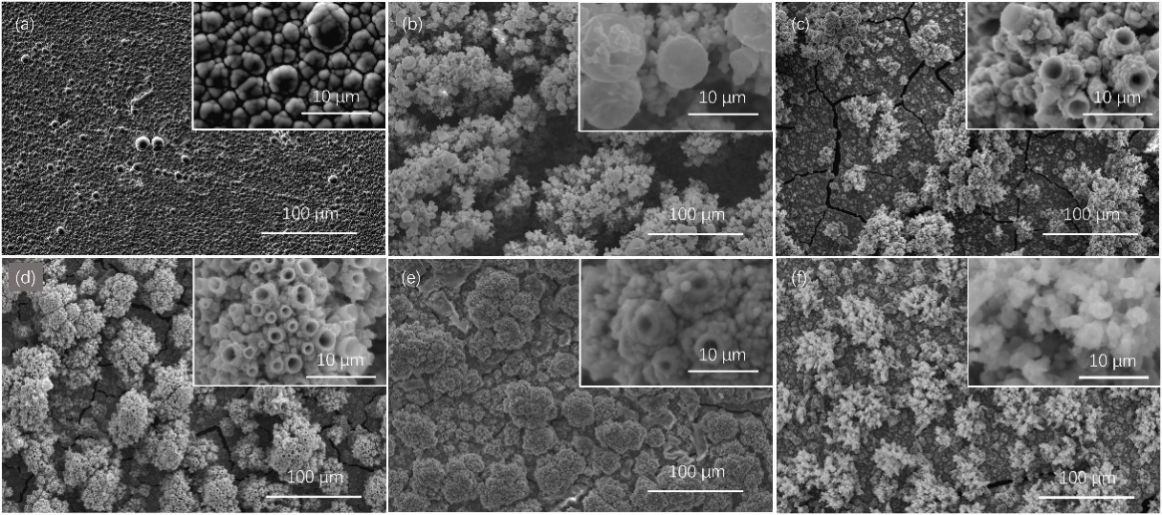
SEM image of electrodeposited Ni_x_S_y_ samples on carbon plate in electrolyte containing 100 mM NiSO_4_, 20 mM NiCl_2_ and 60 mM H_3_BO_3_ under current densities of 5 mA cm^−2^ for 2 h, with CS(NH_2_)_2_ concentration of (a) 0 M, (b) 0.25 M, (c) 0.50 M, (d) 0.75 M, (e) 1.00 M and (f) 1.25 M.

A change in the concentration of thiourea for electrodeposition leads to a change in the product^[56]^ and this can result in different compositions of nickel sulfides, such as NiS, NiS_2_ and Ni_3_S_2_, among which Ni_3_S_2_ should have the best HER performance.^[36]^ Morphology and composition study was performed via SEM and EDS on samples of different thiourea concentrations. As shown in Figure 5, various concentrations of thiourea may lead to different deposited morphology. When no thiourea is added, pure nickel is deposited with a uniform grain distribution, and EDS confirms the deposit to be pure Ni. The deposited nickel shows a dense structure and is crack‐free, firmly attached to the carbon plate. With the addition of thiourea, a porous layer formed on the electrode surface with gathered particles. After adding 0.25 M thiourea, smooth spheres could be found on top of the surface. A coral‐like structure is observed with a thiourea concentration higher than 0.5 M, showing a unique hollow structure at the end of the particle clusters, providing a higher surface area. Also, with a higher thiourea concentration of 0.75–1.00 M, the clusters on top of the substrate seem more evenly distributed, forming channels underneath the particle clusters. With a concentration of 1.25 M, the coral‐like structure is replaced by fine particles. These morphology changes may suggest a shift in composition, indicating thiourea concentration is an essential factor in controlling the final product.

**Figure 6:**
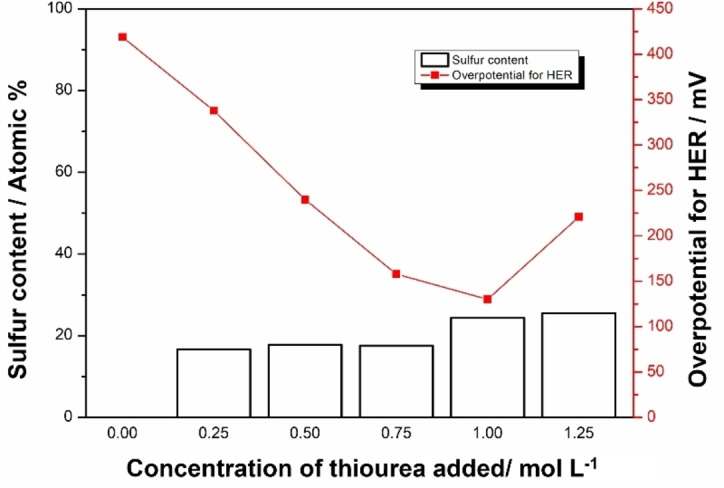
Sulfur content measured by EDS and overpotential for HER of the Ni_x_S_y_ coating prepared with different concentrations of thiourea added in the electrodeposition electrolyte.

As is shown in Figure 6 the change in sulfur content under different concentrations of thiourea can be observed. The sulfur content was obtained via EDS, where point analysis was applied for each sample as is shown in Figure S2. Average sulfur content was then taken into account for an estimation of the actual composition, shown as Table S1. As the concentration of thiourea added to the electrolyte increases, the sulfur content in the sample detected by EDS becomes higher. This trend shows that a higher concentration of thiourea is beneficial for obtaining a higher sulfur content in the deposit. Combined with the overpotential obtained on RDE setup shown in Figure 4(d), a drastic reduction in overpotential for HER at 10 mA cm^−2^ in 1 M KOH can be observed from 420 mV (without thiourea) to 125 mV (with 1 M of thiourea). However, when the thiourea concentration rises to 1.25 M, the overpotential for HER rises to around 225 mV, possibly because of the shedding of the catalyst during the test. Ni_x_S_y_ synthesised under 1.25 M suffered from a more noticeable peel‐off from the substrate during the test, where black particles can be found at the bottom of the cell after the test. In addition, a less coral‐like structure is observed on the sample prepared with a thiourea concentration of 1.25 M from the SEM image in Figure 5(f), which accounts partially for this reduction in performance.

#### 2.2.3 Boric Acid Concentration

Boric acid serves as the buffer during the electrodeposition to help maintain the pH of the solution to be steady, which is used in Watt's Bath.^[45]^ Figure 4(c) displays the LSV curves of catalysts synthesised with different concentrations of boric acid. The buffer helps to resist the local pH change at the surface of the electrode, due to the electrochemical decomposition reaction of [Ni(SC(NH_2_)_2_)_6_]^2+^ (Equation (2)), the generation of H_2_ gas may lead to increased pH and thus the formation of Ni(OH)_2_ and or NiO at the surface of the electrode during deposition,^[58]^ which are not as catalytically active for HER as nickel sulfides.^[50]^ From the LSV results, the addition of boric acid noticeably improves the HER performance of deposited catalysts. However, the difference between the performance of the catalysts synthesised from solutions containing 20 mM, 40 mM, 60 mM, and 80 mM H_3_BO_3_ is not significant. The catalyst synthesised from the solution containing 60 mM boric acid performed slightly better than the rest and was thus chosen as the optimised concentration for the following experiments.

#### 2.2.4 Current Density for Deposition

Figure 4(d) shows polarisation curves for the HER performance of Ni_x_S_y_ catalyst deposited at different current densities, for which the deposition times were adjusted correspondingly to keep the deposition capacity constant. The concentration of NiSO_4_, NiCl_2_, CS(NH_2_)_2_ and H_3_BO_3_ in the deposition electrolyte were kept constant during the experiments. As shown in Figure 4(d), different deposition current densities lead to various HER performance. The best performance comes with the catalyst deposited at −5 mA cm^−2^ for 1200s. The reasons are considered from three perspectives: Firstly, a higher deposition current density results in a larger overpotential applied during the electrodeposition process, which could potentially lead to favoured Ni deposition; a more negative applied potential is required for reaching higher current densities, which may also promote hydrogen evolution as a side reaction during the electrodeposition process of the catalyst, leading to a result of lower Faraday efficiency for catalyst deposition. This partially explains why catalyst deposited at −5 mA cm^−2^ performs better than catalyst deposited at −8 mA cm^−2^ and −10 mA cm^−2^. Secondly, the porosity of the catalysts can be affected by the deposition current density. When a smaller current density is applied, the catalyst layer observed on the surface of the electrode is thinner and denser than the higher current density deposits, that has a lower porosity and surface area; hence, a worse HER performance is expected. This is why catalysts deposited at −2 mA cm^−2^ and −4 mA cm^−2^ are not as desirable as catalyst deposited at −5 mA cm^−2^. Thirdly, the mechanical stability of the catalyst is compromised at higher deposition current densities. When a higher current density is applied, HER is introduced into the deposition process, leading to a highly porous and fragile structure because of the generation and removal of the bubbles, which may cause the catalyst to detach more easily.^[29]^

**Figure 7:**
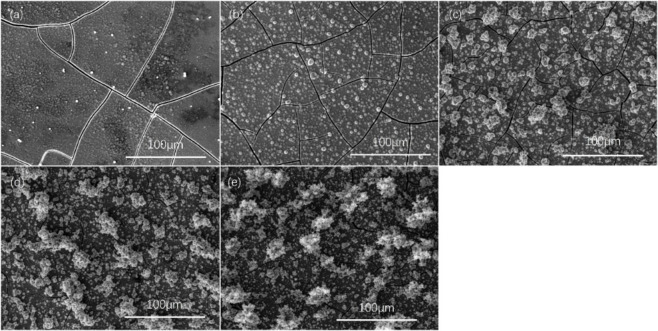
SEM image of electrodeposited Ni_x_S_y_ samples on non‐conductive tape masked 1 cm^2^ carbon plate in electrolyte containing 100 mM NiSO_4_, 20 mM NiCl_2_, 1 M CS(NH_2_)_2_ and 60 mM H_3_BO_3_ under current densities of (a) 1 mA cm^−2^ for 100 min, (b) 3 mA cm^−2^ for 33.3 min, (c) 5 mA cm^−2^ for 20 min, (d) 7 mA cm^−2^ for 14.3 min and (e) 10 mA cm^−2^ for 10 min.

SEM and EDS were used to confirm the morphology and composition of the deposit prepared under different conditions. Figure 7 shows the SEM images captured on the Ni_x_S_y_ film deposited under different current densities. A smooth film was obtained when 1 mA cm^−2^ current density was applied. Small particles are formed on top of a film when 5 mA cm^−2^ was applied. Stacking and clusters of particles are formed on top of the thin film was noticeable with current densities are higher than 7 mA cm^−2^. A higher current density is favoured for its rapid deposition rate, and would generate a rougher surface with particles attached, which could be beneficial for providing higher surface area; Figure S3 and Table S2 shows the trend of sulfur content change in the deposit prepared under different current densities, where higher sulfur content can be found when lower current densities were applied. As suggested in Figure 1, this trend could be from the preferred deposition of Ni metal at higher current densities. Combined with the result shown in Figure 4(d), the performance of the catalysts prepared under current densities between 1–5 mA cm^−2^ appears to be improving while performance appeared to decrease for catalysts prepared under 5–10 mA cm^−2^, which was an effect of both morphology and sulfur content. The use of a current density of 5 mA cm^−2^ showed a good balance between them, leading to the best performance.

Based on the above results, concentration of thiourea and current density are the most influential factors. From the results obtained in this section, the optimised catalyst was that prepared from an electrolyte containing 100 mM NiSO_4_ and 20 mM NiCl_2_ as a source of nickel, 1 M CS(NH_2_)_2_ as a source of sulfur, 60 mM H_3_BO_3_ as a buffer and deposited at 5 mA cm^−2^ for 1200 s.

### 2.3 Electrodeposition of Ni_x_S_y_ Catalyst on Microelectrode Setup

**Figure 8:**
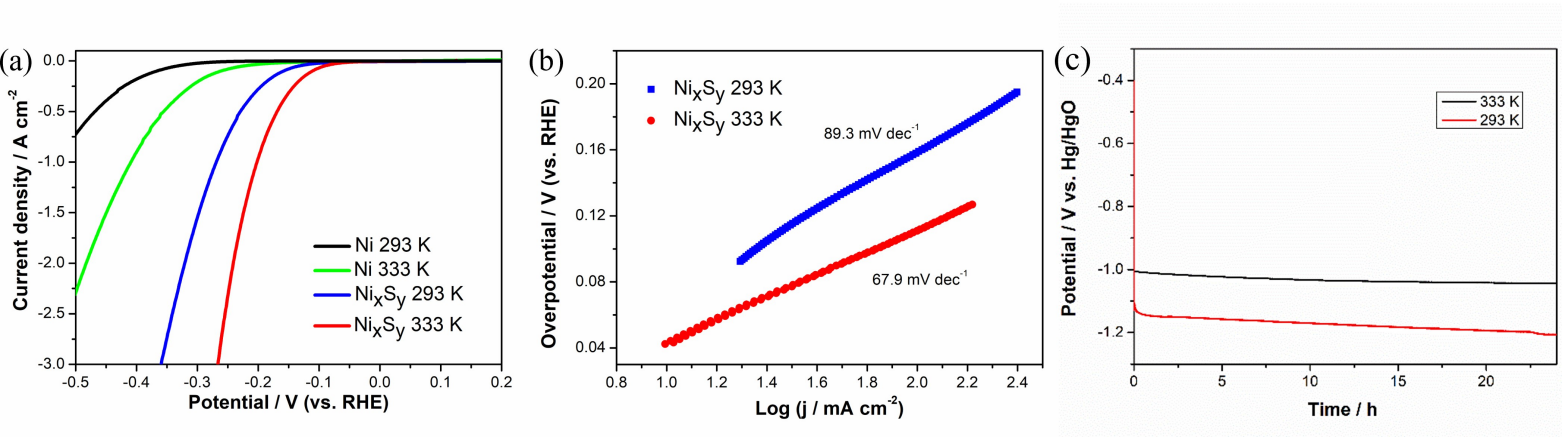
(a) Polarisation curve recorded using linear sweep voltammetry obtained on bare Ni microelectrode with a diameter of 50 μm at a scan rate of 20 mV s^−1^ at 293 K, 333 K, and Ni_x_S_y_ catalyst coated Ni microelectrode at 293 K and 333 K without IR compensation. (b) Tafel slope for Ni_x_S_y_ coated Ni microelectrode under 293 K and 333 K in 1 M KOH; (c) E−t curve recorded for HER on Ni_x_S_y_ coated Ni microelectrode in 1 M KOH solution at 100 mA cm^−2^ current density for 24 h under 293 K and 333 K, respectively.

The non‐exponential shape of the LSVs obtained during RDE HER testing (Figure 4) indicates that there is a significant Ohmic drop in the RDE setup, which makes testing at high current densities such as 1 A cm^−2^ difficult. The ohmic drop is likely to come from the resistance generated by bubble formation and mass transportation. To further reduce the influence of IR drop introduced by the setup and focus more on the catalytic activity of the deposited sample under higher current densities, the electrochemical performance was studied using a microelectrode setup. A microelectrode setup has the advantage of a tiny electrode surface area (approx. 1.963×10^−5^ cm^2^), only an extremely small current is required to reach higher current densities, thus lowering the system's IR drop. Figure 8(a) shows the LSV curves on the polished Ni microelectrode and Ni_x_S_y_ coated Ni microelectrode in 1 M KOH at 293 K and 333 K respectively. All the LSV results are presented without IR compensation, which is negligible. Tafel slope of the Ni_x_S_y_ was also calculated from the LSV obtained, as is shown in Figure 8(b), 67.9 mV dec^−1^ and 89.3 mV dec^−1^ for 333 K and 293 K. Table 1 shows the HER overpotential of the four samples under different current densities, as taken from Figure 8. Coating with a catalyst has dramatically reduced the overpotential needed for HER, compared to those without coating. To achieve HER at a current density of 10 mA cm^−2^, the Ni microelectrode without catalyst coating requires 265 mV and 174 mV at 293 K and 333 K, respectively, while the electrode coated with Ni_x_S_y_ only requires 45 mV and 41 mV under the same conditions. For circumstances at higher current densities, taking the overpotential at 500 mA cm^−2^ as an example: a bare Ni microelectrode needs 469 mV of overpotential at 293 K and 373 mV at 333 K, while for the Ni_x_S_y_ coated Ni electrode only requires 230 mV and 169 mV, respectively, which shows an improvement of 239 mV and 204 mV by coating with Ni_x_S_y_. This result shows the excellent performance of the deposited catalyst. Double layer capacity of the non‐faradic region was also recorded using CV at different scan rates, as is shown in Figure S4. Compared between the catalyst coated and uncoated electrodes, the capacitance increased by 10 %, which suggests an increase in electrochemical active surface area (ECSA) of 10 %. The minor augmentation of ECSA on the coated electrode indicates that the improvement in HER performance is due predominantly to the catalytic activity of Ni_x_S_y_, rather than enhanced surface area.

**Table 1:** Overpotential for HER measured in 1 M KOH on Ni microelectrode with and without Ni_x_S_y_ coating tested at 293 K and 333 K recorded from LSV at a scan rate of 20 mV s^−1^.

Catalyst	Overpotential for HER/mV					
	10 mA cm^−2^	20 mA cm^−2^	50 mA cm^−2^	100 mA cm^−2^	500 mA cm^−2^	1 A cm^−2^
Uncoated Ni ME 293 K	265	294	333	366	469	529
Uncoated Ni ME 333 K	174	211	255	288	373	425
Ni_x_S_y_ coated Ni ME 293 K	45	94	134	158	230	269
Ni_x_S_y_ coated Ni ME 333 K	41	65	90	111	169	202

Chronopotentiometry (CP) was also used to assess the overpotential change during a 300 s period of electrolysis under different current densities. As Figure S5 shows, at low current densities the overpotential increases rapidly at the beginning of each CP, reaching a steady stage where the potential stays relatively stable. Under higher current density situations, the curve becomes noisier and usually tends to show a linearly increasing overpotential instead of staying constant. This can be attributed to a faster rate of bubble generation, prior to departure from the surface of the electrode, blocking the active sites and thus impacting the system's resistance and this is reflected in noisier potential responses and larger overpotentials. The recorded data is presented in Table 2, which shows the overpotential at the end of each CP test. Compared to the result obtained from LSV shown in Table 1, albeit at low current densities the overpotential reading is approximately the same from both methods, voltages collected at higher current densities from CP under steady state conditions, are usually higher than those obtained by LSV under higher current densities. Because HER is a gas evolution process, the fluctuation of the curve recorded by CP at a very high current density at 10 A cm^−2^ can be obviously seen, which shows the impact of the generation and removal of the bubbles. Between these two techniques, CP is more analogous to actual working conditions than LSV, and therefore more practical for providing operational overpotential data.

**Table 2:** Overpotential for HER tested of Ni microelectrode with and without Ni_x_S_y_ coating under 293 K and 333 K in 1 M KOH obtained with chronopotentiometry method.

Catalyst	Overpotential for HER/mV					
	10 mA cm^−2^	20 mA cm^−2^	50 mA cm^−2^	100 mA cm^−2^	500 mA cm^−2^	1 A cm^−2^
Uncoated Ni ME 293 K	282	309	359	412	536	628
Uncoated Ni ME 333 K	194	236	294	336	474	546
Ni_x_S_y_ coated Ni ME 293 K	94	119	159	200	313	404
Ni_x_S_y_ coated Ni ME 333 K	47	64	88	112	200	264

Long‐term stability test was carried out on a Ni microelectrode with Ni_x_S_y_ coating. Figure 8(c) shows the E‐t curve recorded during the 24 hour HER in 1 M KOH at 293 K and 333 K, respectively. The design of the microelectrode setup facilitates the release of gas generated during the operation upwards through the electrolyte. The catalyst showed good stability with the overpotential staying relatively steady. The initial overpotentials for the prepared catalyst for HER at 100 mA cm^−2^ are 209 mV and 80 mV at the beginning and 280 mV and 117 mV after the 24 h operation under 293 K and 333 K, respectively. Although the microelectrode holds the ability to reach high current densities with low IR in the system, gas evolution still affects the performance. Different from a full cell situation, where flowing electrolyte introduces mechanical forced convection, removing the bubbles more effectively, micro bubbles are also generated on microelectrode and harder to be removed than regular sized macro bubbles. These reasons account for the increased cell overpotentials.

### 2.4 Full Cell Experiment

**Figure 9:**
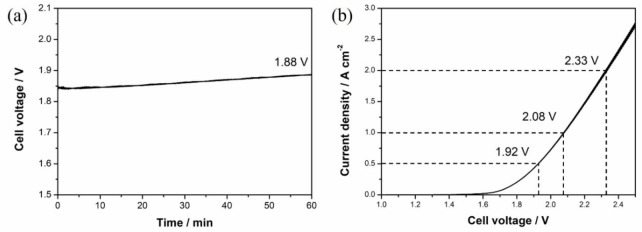
Water electrolysis of AEM full cell with Ni_x_S_y_ coated Ni mesh as cathode and NiFe(OH)_2_ coated Ni mesh as anode, (a) E−t curve recorded by chronopotentiometry while applying 2 A (500 mA cm^−2^) for 1 h, (b) I−E curve recorded on the AEM cell using linear sweep voltammetry with scan rate of 20 mV s^−1^.

To further investigate the performance of Ni_x_S_y_ HER catalyst, tests in the zero‐gap full AEM cell were carried out with NiFe(OH)_2_ as anode material, which has been widely reported as an excellent oxygen evolution reaction (OER) catalyst in alkaline environment.^[59]^ Steady cell voltage can be observed through the 1 h period electrolysis at 500 mA cm^−2^, as shown in Figure 9(a), where the cell voltage was at around 1.85 V to 1.88 V to maintain the current density. The slightly increased potential may come from the increase of the IR drop and the blocking of active sites of the catalysts due to the gas generation. LSV was also used to check the performance of the cell under different potentials after the 1 h testing. As shown in Figure 9(b), 1.92 V, 2.08 V and 2.33 V were required to achieve the current density of 0.5 A cm^−2^, 1.0 A cm^−2^ and 2.0 A cm^−2^ respectively. The reason for the slight decrease in performance comes from the abrasive force introduced by forced convection of the electrolyte, where the catalyst has been removed under such high flow rate (250 mL min^−1^). SEM was used to check the status of the catalyst coating before and after the electrolysis, as is shown in Figure S6, where damaged coating of the catalyst can be found after electrolysis. Some catalysts were found attached to the membrane, which could also account for part of the coating loss. Further work on improving the stability of the catalyst layer is needed.

Table 3 shows a summary of some similar work of AEM water electrolysis performance in the literatures. PGM‐based catalysts tend to have a higher catalytic activity than non‐PGM ones. Comparatively, our present AEM cell performance showed lower cell voltage than most of the cells with PGM‐free catalysts, and even comparable to those with PGM‐based catalysts. It is expected to further improve the performance of the cell by introduction of gas diffusion layer and ionomers in our cell.

**Table 3:** Summary of some full AEM water electrolysis performance reported in literature.

Cathode	Anode	Membrane	Temperature	Electrolyte	Cell voltage	Ref
Ni_x_S_y_ on Ni mesh	NiFe(OH)_2_ on Ni mesh	FAA‐3‐30	333 K	1 M KOH	500 mA cm^−2^ at 1.88 V	This work
Pt black on carbon paper	IrO_2_ on Ti foam	Tokuyama A 201	323 K	1 M KOH	480 mA cm^−2^ at 1.80 V	^[60]^
Pt/C on carbon paper	IrO_2_ on Ti paper	FAA‐3‐50	323 K	1 M KOH	1 A cm^−2^ at 1.80 V	^[61]^
Pt/C	NiMn_2_O_4_	FAA‐3‐50	353 K	1 M KOH	530 mA cm^−2^ at 2 V	^[62]^
Pt	IrO_2_	Sustainion X37‐50	333 K	1 M KOH	1 A cm^−2^ at 1.63 V	^[63]^
NiFeCo	NiFe	Sustainion X37‐50	333 K	1 M KOH	1 A cm^−2^ at 1.9 V	^[63]^
Pt/C	IrO_2_	PFTP‐13	353 K	1 M KOH	7.68 A cm^−2^ at 2.00 V	^[64]^
Ni‐Fe	Ni‐Fe	PFTP‐13	353 K	1 M KOH	1.6 A cm^−2^ at 2.00 V	^[64]^
3‐Co_3_S_4_ NS/NF	CO_3_S_4_	Sustainion X37‐50	318 K	1 M KOH	431 mA cm^−2^ at 2.00 V	^[65]^

### 2.5 Physical Characterisation

**Figure 10:**
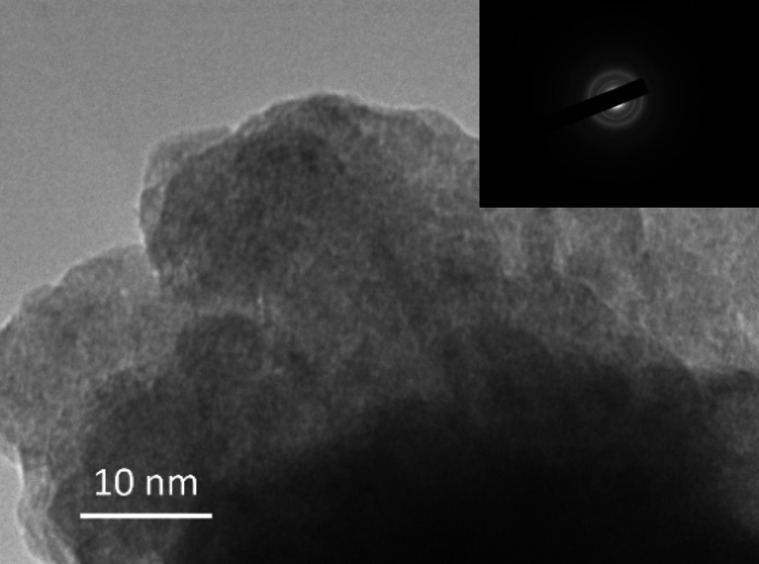
TEM image and the SAED image obtained on the exfoliated electrodeposited Ni_x_S_y_ film.

XRD was undertaken to obtain information on the composition and crystallinity of the deposits with the optimised recipe. As is shown in the XRD patterns in Figure S7, no peaks associated with nickel sulfide are found in the spectrum; only the Ni peaks from the metal substrate were presented. Also, the increasing thickness of the deposit layer reduces the intensity of Ni diffraction peaks from the substrate, indicating the amorphousness of the deposited layer. Figure 10 shows the TEM image and the selected area diffraction (SAED) image (inset). From the high magnification image, no clear crystal structure was observed. The diffraction ring pattern of the SAED image suggests the deposit to be amorphous;^[66]^ amorphous materials do not have a fixed crystalline structures, granting them structural flexibility, higher electrochemical surface area and better stability.^[53]^ Self‐healing of the catalysts during operation also becomes feasible with this flexible feature.

**Figure 11:**
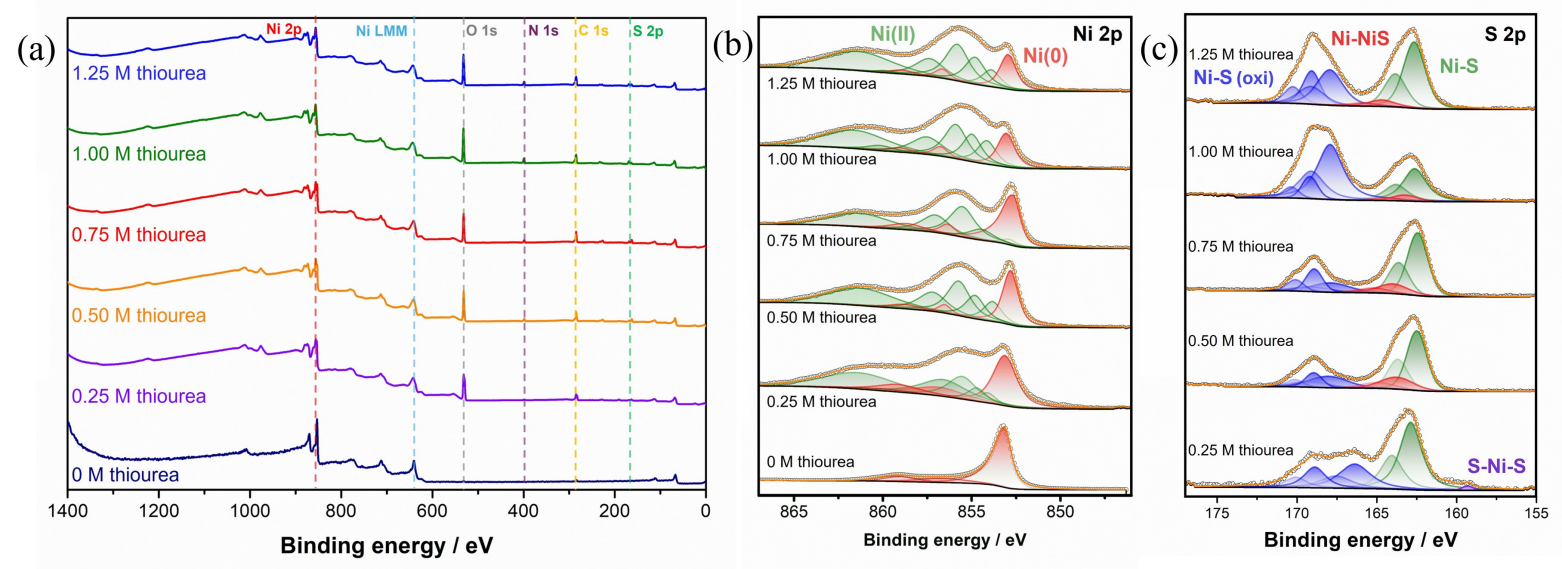
(a) Survey scan of the XPS spectrum obtained on samples prepared with different thiourea addition. And fitting result of XPS data on high resolution scan of (b) Ni 2*p* and (c) S 2*p* orbitals.

XPS was used to gain further information on the bonding and status of Ni and S elements of the catalyst's surface. Samples with different concentrations of thiourea in electrodeposition electrolyte were prepared on polished carbon plates. Figure 11 shows the survey XPS spectrum.

Both Ni and S are detected in the samples prepared with the addition of thiourea; no trace of S was found in the sample synthesised without. A binding energy shift of Ni 2*p* was found from 853 eV for pure Ni to 854.5–855.9 eV after the formation of Ni_x_S_y_. The average electronegativity of S^2−^ as a ligand lies between Br^−^ and Cl^−^, at around 2.9. A binding energy of around 855.5 eV, according to the fitting result^[67]^ between Ni and S, is expected, which matches the result obtained in this study, indicating the formation of nickel sulfide. An Auger peak at 846.05 eV was also detected, which is shown as Ni LMM in Figure 11 and Figure S8. This Auger peak is for pure Ni sample without thiourea, while kinetic energy of around 842.00 eV were found for Auger peaks obtained on samples prepared with addition of thiourea.

Fitting of the high‐resolution scan peaks of Ni 2*p* 3/2 and S 2*p* was also carried out. Satellite peaks of 2*p* 3/2 can be found in high resolution Ni 2*p* scan (Figure 11) with a difference of binding energy of 6 eV, which is attributed to a predominant surface plasmon loss.^[67]^ For Ni metal, according to the literature, the core line for Ni 2*p* 3/2 is at 852.4 eV,[^29^, ^68^] and here we used multiplets to better fit the actual Ni status. All of the samples show a Ni(0) peak (at around 853.0 eV), which indicates the deposit is a combination of Ni metal and sulfides. A higher binding energy for Ni 2*p* indicates the surface of the deposit to be a sulfide compound.^[69]^ Comparing these data, the main peaks of Ni 2*p* 3/2 at around 854.5–855.5 eV, can be assigned to nickel sulfide.^[29]^ A mixture of multivalence oxidised Ni is formed because of different compounds formed between Ni and S.^[70]^ Compared with the data base in National Institute of Standards and Technology (NIST), the observed binding energy of Ni peak and S peak can match NiS according to previous literature.^[71]^ As the concentration of thiourea increases, a higher Ni−S content can be found in the sample, which suggests the same trend as in Figure 6. The composition between 1.25 M and 1 M thiourea addition are very similar. Combined with the electrochemical tests, a higher Ni−S content is favourable for a better catalytic activity for HER in alkaline.

The high‐resolution scan of S 2*p* is shown in Figure 11. The data was normalised based on the minimum and maximum reading to better focus on the shift of binding energy instead of the relative intensity of the peaks. Peaks were assigned to S 2*p* 3/2 and 2*p* 1/2 with area controlled to be in 2 : 1 ratio and energy difference of 1.18 eV. Compared with the data base and previous literature, the S 2*p* 3/2 peak at around 164 eV and 162 eV could be assigned to Ni_3_S_2_ and NiS, respectively. It is also possible for the peaks at 164 eV to be assigned for polysulfide with central S, and the peak with lower binding energy at around 162 eV should be polysulfide with terminal S.^[72]^ The appearance of Ni‐NiS in higher thiourea concentration conditions could result from the deposition of Ni_3_S_2_, which is beneficial for improving the electrochemical performance. Peaks found in range of 167–170 eV should be assigned to the oxidised Ni−S.^[71]^ Since XPS is a superficial technique, which is only capable of collecting surface information, oxidation may have occurred at the surface due to the exposure to air.

From the wide scan shown in Figure 11, with the concentrations of thiourea higher than 0.50 M, the appearance of N is also observed, which may indicate the doping of N to the surface of the catalyst. This process may happen due to the reduction of NH_4_^+^ formed from decomposition of CO(NH_2_)_2_ or CS(NH_2_)_2_. The intensity of the peak gets stronger as the concentration of thiourea increases, suggesting thiourea serves as a N source, which has been reported in other literature via thermal decomposition method to form N‐doped nickel sulfide.^[73]^ N‐doping has been used in studies of the development of water splitting catalysts to optimise their electronic structure of the catalysts based on experimental and simulated results.[^73^, ^74^, ^75^] Combined with the HER performance estimated via previous experiments shown in Figure 4(b), N‐doping could be part of the reason for improved performance at higher thiourea addition conditions.^[73]^

## 3 Conclusions

In this study, the electrodeposition of amorphous Ni_x_S_y_ is systematically studied. Thiourea concentration and deposit current density are proved to be the most important parameters for the optimised morphology of Ni_x_S_y_ and thus its electrochemical HER catalysis performance. The optimised recipe for Ni_x_S_y_ deposition is 100 mM NiSO_4_, 20 mM NiCl_2_, 1.00 M thiourea, and 60 mM H_3_BO_3_ with a cathodic current density of 5 mA cm^−2^ for 1200 s. Physical characterisation confirms the amorphous feature of the deposit, and XPS results suggest N‐doping happens under high thiourea concentration conditions, which is beneficial for improving the HER catalytic activity of nickel sulfide catalyst. To achieve HER at a current density of 500 mA cm^−2^, 169 mV of overpotential is required, with the Ni_x_S_y_ coated microelectrode under 333 K. Long‐term stability tests were carried out on microelectrode setup, where the catalyst showed steady performance at a current density of 100 mA cm^−2^ at the beginning of electrolysis over 24 h with overpotentials of only 80 mV under 333 K, which became 117 mV after the operation. Full AEM cell testing approved the practical application of the optimised Ni_x_S_y_ catalyst, achieving 500 mA cm^−2^ at 1.88 V under 333 K, suggesting it to be an excellent HER catalyst for AEM water electrolysis.

## Conflict of Interests

The authors declare that they have no known competing financial interests or personal relationships that could have appeared to influence the work reported in this paper.

4

## Supporting information

As a service to our authors and readers, this journal provides supporting information supplied by the authors. Such materials are peer reviewed and may be re‐organized for online delivery, but are not copy‐edited or typeset. Technical support issues arising from supporting information (other than missing files) should be addressed to the authors.

Supporting Information

## Data Availability

The data that support the findings of this study are available from the corresponding author upon reasonable request.
