# Supplementary material for: Electrodeposition and Optimisation of Amorphous NixSy Catalyst for Hydrogen Evolution Reaction in Alkaline Environment
Source: Chemistry. 2024 Nov 7;30(66):e202403030. doi: 10.1002/chem.202403030 (PMC11590175; doi:10.1002/chem.202403030)
Supplement: Supplementary file 1 — Supporting Information [file CHEM-30-e202403030-s001.pdf]

Chemistry–A European Journal

Supporting Information

Electrodeposition and Optimisation of Amorphous Ni_xS_y Catalyst for Hydrogen Evolution Reaction in Alkaline Environment

Cheng Lyu, Adeline Loh, Mikey Jones, David Trudgeon, Jack Corbin, Jianyun Cao, Zhenyu Zhang, Peter Connor, and Xiaohong Li*

Supporting information for

ELECTRODEPOSITION AND OPTIMISATION OF AMORPHOUS NI_XS_Y CATALYST FOR HYDROGEN EVOLUTION REACTION IN ALKALINE ENVIRONMENT

Cheng Lyu [a], Adeline Loh [a], Mikey Jones [a], David Trudgeon [b], Jack Corbin [a], Jianyun Cao [c], Zhenyu Zhang [a], Peter Connor [a], Xiaohong Li *[a]

[a] *Renewable Energy Group, Department of Engineering, Faculty of Environment, Science and Economy, University of Exeter, Penryn Campus, Cornwall TR10 9FE, UK*

[b] *Camborne School of Mines, Department of Earth and Environmental Science, Faculty of Environment, Science and Economy, University of Exeter, Cornwall TR10 9FE, UK*

[c] *Yunnan Key Laboratory of Electromagnetic Materials and Devices, National Center for International Research on Photoelectric and Energy Materials, School of Materials and Energy, Yunnan University, China*

* *Corresponding author.*

E-mail: X.li@exeter.ac.uk

1. Electrochemical measurements

Nernst equation was used to calculate the potential for Ni^{2+}/Ni as follows:

$$E = E^0 + 2.303\frac{RT}{nF}\log\alpha(Ni^{2+}) \qquad \text{Equation (S1)}$$

Where the standard potential for nickel deposition, is -0.257 V vs SHE, the Ag/AgCl vs SHE is +0.197 V under standard conditions (1 atm, 25°C), and the concentration of Ni^{2+} in the solution is 100 mM.

All the measured potentials corresponding to the Hg/HgO reference electrode were converted to potentials with respect to the reversable hydrogen electrode (RHE) by using the Nernst equation (Equation S2):

$$E_{\mathrm{RHE}} = E_{\mathrm{Hg/HgO}} + 0.0591\mathrm{pH} + E^0_{\mathrm{Hg/HgO}} \qquad \text{Equation (S2)}$$

where E_{RHE} is the potential in RHE scale, $E_{\mathrm{Hg/HgO}}$ is the measured potential against the Hg/HgO reference electrode, $E^0_{\mathrm{Hg/HgO}}$ = 0.197 V at 25 °C.

2. Physical characterisation

X-ray diffraction (XRD) was carried out on the prepared samples to study the composition and crystal structural information using the Bruker D8 advanced XRD. An extended period of electrodeposition with the same recipe was carried out for the sample preparation to improve the signal, the sample was prepared under chronopotentiometry at a cathodic current density of 5 mA cm^{-2} for 2 h. A Ni foil was used as the substrate, and the surface area was controlled using insulating tape. Results were further analysed using DIFFRAC EVA, software and database provided by Bruker. **Figure S1** shows the XRD pattern obtained on the thick deposited Ni_xS_y (2 h deposition time) and of thin deposited Ni_xS_y (20 min deposition time) on Ni foil, where only Ni peak is found, no nickel sulfide peaks were found, indicating the deposit to be amorphous.

Figure S2 shows the Auger peak of Ni from the XPS result obtained on Ni_xS_y samples prepared under different thiourea concentrations. A shift in the Ni LMM peak indicates the change of Ni valance from 0 to +2.

3. Supplementary Figures

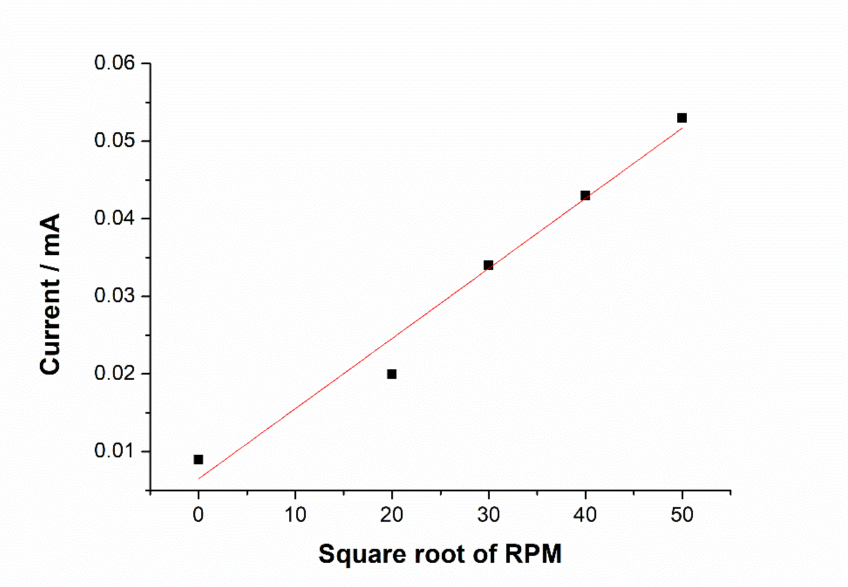

Figure S1 Current vs square root of RPM at -0.56 V vs Ag/AgCl in 100 mM $NiSO_4$, 20 mM $NiCl_2$, 60 mM H_3BO_3 and 1 M $CS(NH_2)_2$ solutions with pH controlled to 4 by adjusting with 0.01 M H_2SO_4.

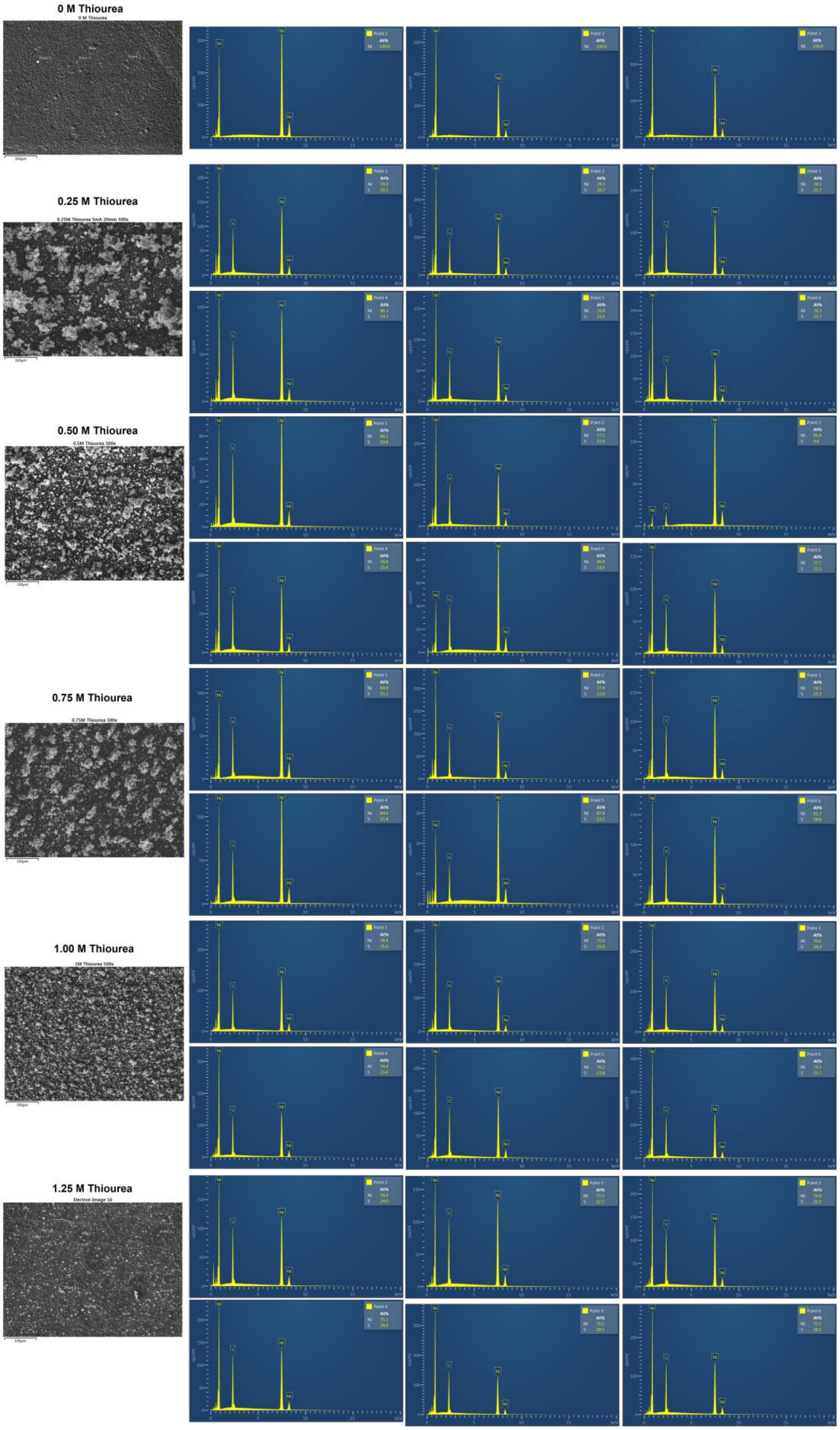

Figure S2 EDS data collected on Ni_xS_y samples prepared with different concentrations of thiourea.

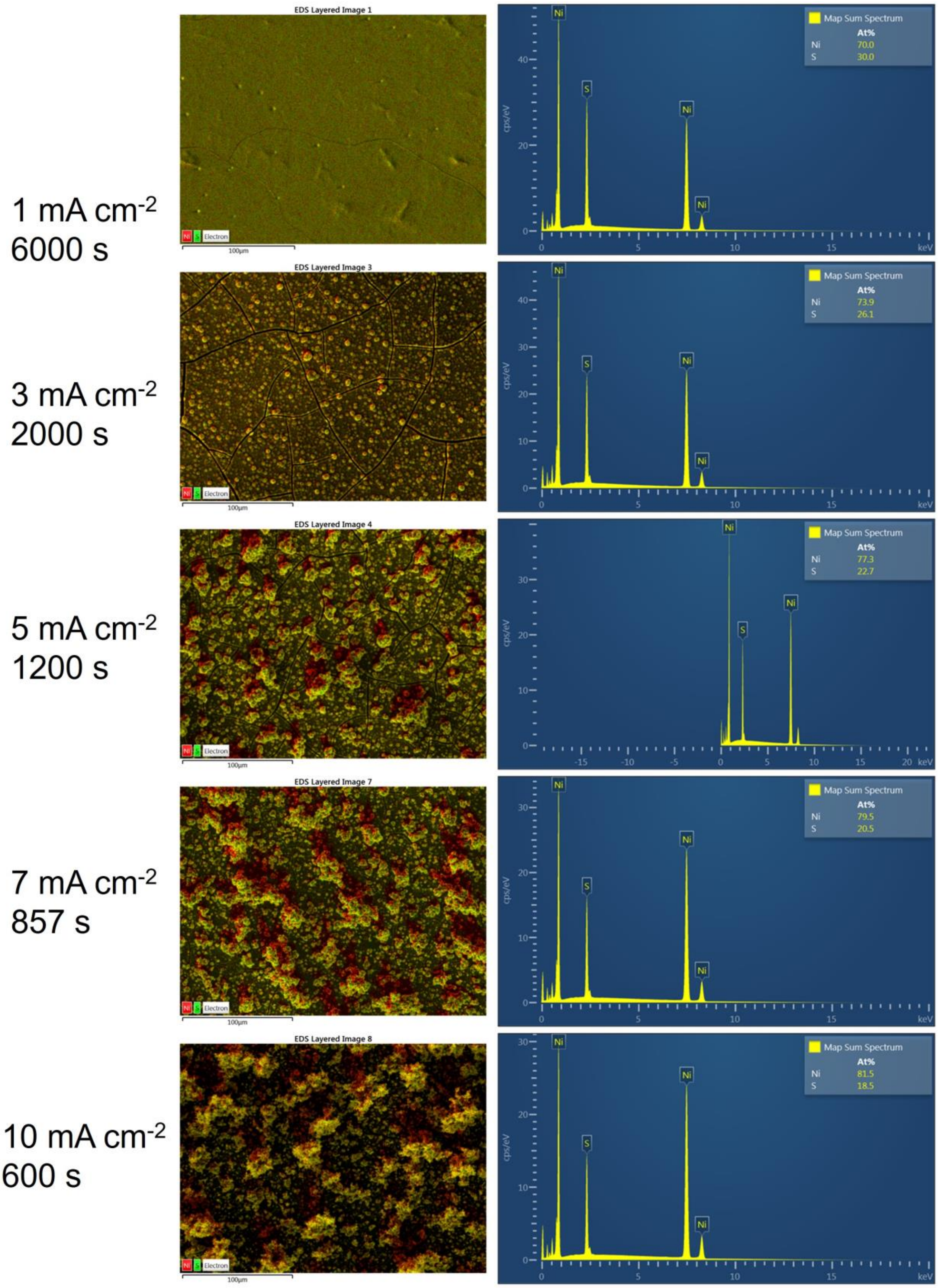

Figure S3 EDS data collected on Ni_xS_y samples prepared under different current densities with same amount of electron transferred via CP method.

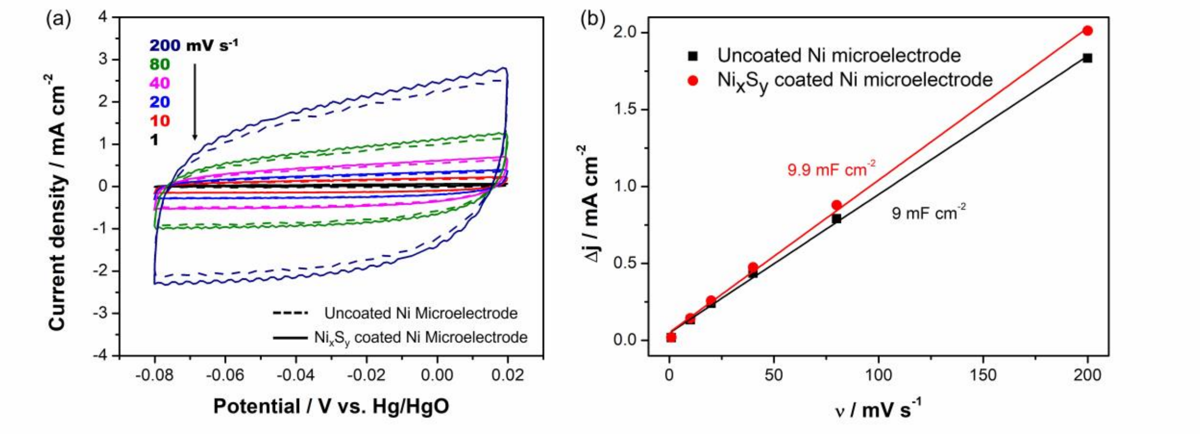

Figure S4 (a) Cyclic voltammograms of Ni_xS_y coated and uncoated Ni microelectrode (D = 50 μm) in 1 M KOH under scan rate of 1,10,20,40,80 and 200 mV s^{-1} in range of –0.08 V to 0.02 V vs. Hg/HgO. (b) linear fit of Δj ($\Delta j = (j_a - j_c)/2$) vs. scan rate. The Ni_xS_y is coated at 5 mA cm^{-2} current density for 1200 s with the optimised deposition recipe.

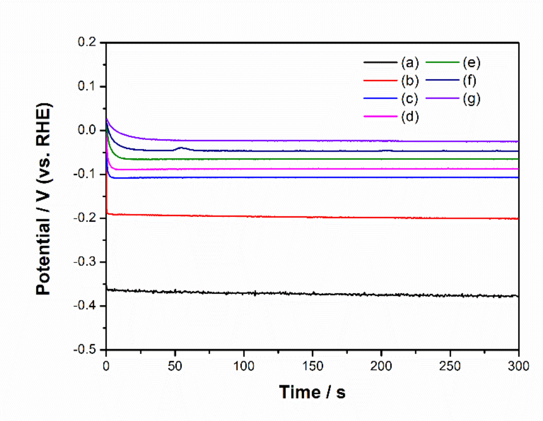

Figure S5 V-t curve recorded by CP at a current density of (a) 10 A cm^{-2}, (b) 1 A cm^{-2}, (c) 100 mA cm^{-2}, (d) 50 mA cm^{-2}, (e) 20 mA cm^{-2}, (f) 10 mA cm^{-2}, (g) 5 mA cm^{-2} over 300 s each, with Ni-S catalyst deposited on Ni microelectrode.

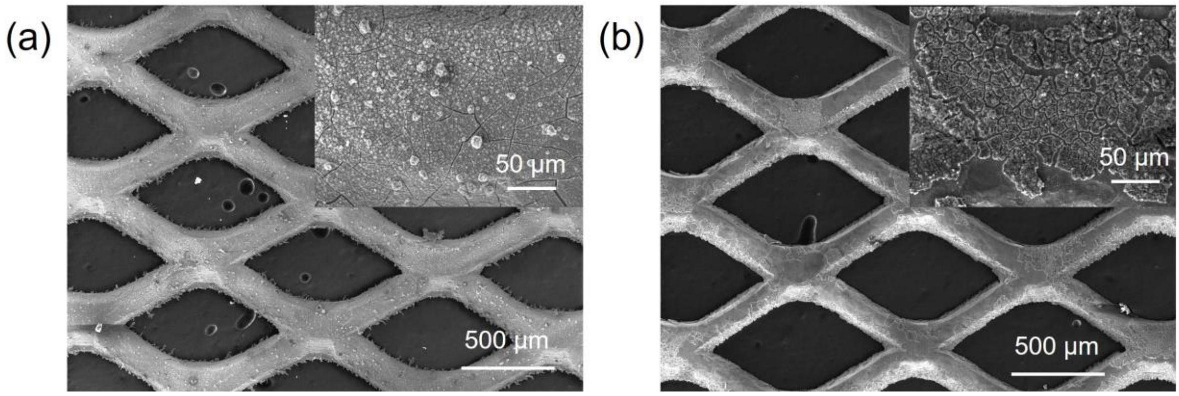

Figure S6 SEM image of Ni_xS_y coated Ni mesh (a) before and (b) after 20 h electrolysis in AEM water electrolyser.

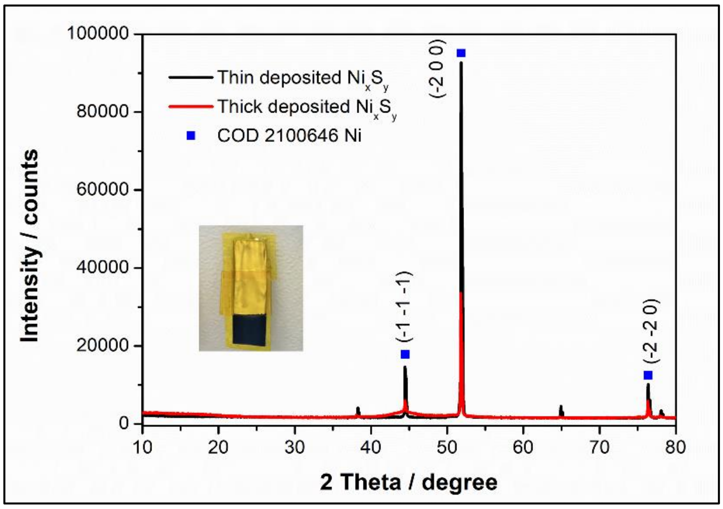

Figure S7 XRD patterns of thick deposited Ni_xS_y (2 h deposition time) and of thin deposited Ni_xS_y (20 min deposition time) on Ni foil.

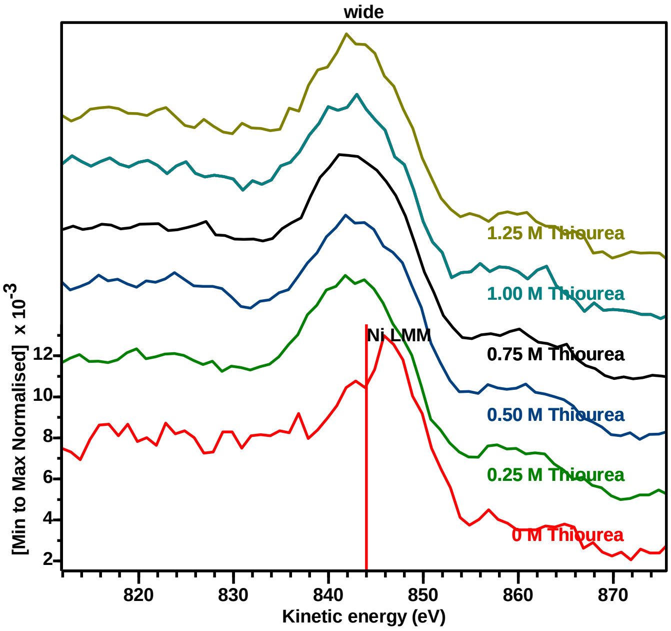

Figure S8 Auger peak of Ni from wide scan of XPS pattern obtained on Ni_xS_y with deposited with different concentrations of thiourea.

4. Supplementary Tables

Table S1 Average sulfur content from EDS data collected on Ni_xS_y samples prepared with different concentrations of thiourea.

Thiourea concentration / M	Sulfur content / at. %
0	0.0
0.25	16.7
0.50	17.8
0.75	17.6
1.00	24.35
1.25	25.4

Table S2 Average sulfur content from EDS data collected on Ni_xS_y samples prepared under different current densities with same amount of electron transferred via CP method.

Current density / mA cm^{-2}	Sulfur content / at. %
1	30.0
3	26.1
5	22.7
7	20.5
10	18.5
